# Developmental Alcohol and Circadian Clock Function

**Published:** 2001

**Authors:** David J. Earnest, Wei-Jung A. Chen, James R. West

**Affiliations:** David J. Earnest, Ph.D., is an associate professor, Wei-Jung A. Chen, Ph.D., is an assistant professor, and James R. West, Ph.D., is a professor in the Department of Human Anatomy and Medical Neurobiology, Texas A&M University Health Science Center, College of Medicine, College Station, Texas

**Keywords:** circadian rhythm, sleep disorder, fetal alcohol effects, prenatal alcohol exposure, fetal alcohol syndrome, neuropeptides, alcohol-related neurodevelopmental disorder, cytolysis, nerve growth factors, CNS nuclei

## Abstract

Studies in rats found that alcohol exposure during the early postnatal period, particularly during the brain-growth-spurt period, can result in cell loss in various brain regions and persistent behavioral impairments. Some investigators have speculated that the body’s internal clock, which is located in the suprachiasmatic nuclei (SCN) in the brain, may also be affected by developmental alcohol exposure. For example, alcohol-induced damage to the SCN cells and their function could result in disturbances of the circadian timekeeping function, and these disturbances might contribute to the behavioral impairments and affective disorders observed in people prenatally exposed to alcohol. Preliminary findings of studies conducted in rats suggest that developmental alcohol exposure may indeed interfere with circadian clock function as evidenced by a shortened circadian sleep-wake cycle and changes in the release of certain brain chemicals (i.e., neuropeptides) by SCN cells.

Since ancient Roman times, people have suspected that maternal alcohol consumption has deleterious effects on the developing fetus. The clinical diagnosis of fetal alcohol syndrome (FAS), however, was not defined until 1973 when Jones and Smith first documented a constellation of characteristics in the offspring of mothers who had abused alcohol during pregnancy. These characteristics include a set of unique facial features, such as a flat groove between the nose and upper lip (i.e., a flat philtrum), a thin upper lip, and a small nose, as well as central nervous system (CNS) dysfunction. The CNS defects—which include reduced brain size (i.e., microencephaly), reductions in the volume of various brain regions (e.g., in the anterior cerebellum^1^ and the corpus callosum^2^) and behavioral impairments (e.g., learning difficulties and attention deficits) (Roebuck et al. 1999)—are by far the most debilitating features associated with FAS (Stratton et al. 1996).

During the past 20 years, studies using animal model systems have generated substantial information on the risk factors associated with maternal alcohol use and the spectrum of alcohol-induced brain injuries among affected offspring. Perhaps most importantly, these animal studies have identified the brain-growth-spurt period as the developmental stage most vulnerable to alcohol-induced brain injury. In humans, this particular brain-growth period begins during the third trimester of pregnancy, peaks around birth, and persists through the first few years of life. In rats, which are the most commonly used species in fetal alcohol research, the corresponding rapid brain-growth period occurs during the first 2 weeks after birth.

Alcohol exposure in rats during this early postnatal period produces structural changes in the brain comparable to those observed in human FAS patients. These changes range from reductions in the rat’s brain size and weight (Maier et al. 1996) to the loss of various cell populations in discrete brain regions, such as the cerebellum and the hippocampus^3^ (Bonthius and West 1990). In addition to these structural changes, developmental alcohol exposure induces a wide spectrum of neurochemical changes in the developing rat brain (Manteuffel 1996).

One key question in the fetal alcohol research field is whether developmental alcohol exposure has long-term consequences. In humans, the characteristic facial abnormalities associated with FAS typically become less prominent with age. However, many reported findings suggest that alcohol-induced deficits in brain development may persist into adulthood. Using noninvasive techniques (e.g., magnetic resonance imaging and positron emission tomography) in a small number of children with FAS, researchers have confirmed brain damage from prenatal alcohol exposure and have determined the related behavioral consequences, including learning deficits, hyperactivity, lack of inhibition, depression, and sleep disturbances (Hannigan et al. 1995; Roebuck et al. 1999).

Studies using animal models have been invaluable in establishing the association between structural brain damage and long-term behavioral impairments. For example, researchers have linked the cell loss that occurs in a rat’s cerebellum and hippocampus from early postnatal alcohol exposure with deficits in motor performance (Thomas et al. 1996) and in learning and memory (Goodlett et al. 1987), respectively. These experimental data could provide a focus for long-term clinical investigations in humans, because some of the FAS patients identified and followed since 1973 have now reached adulthood. Thus, researchers could use noninvasive techniques to determine potential structural brain damage in FAS patients and correlate the findings with adult behavioral impairments.

A recent and still highly speculative area of research focuses on whether prenatal alcohol exposure also has long-term effects on the regulation of the body’s internal clock and whether these potential effects could contribute to some of the previously mentioned behavioral problems associated with FAS. This article first provides a brief review of the structure and function of this internal clock. It then discusses the potential relationship between prenatal alcohol exposure and disturbances in the internal clock, including preliminary results of animal studies assessing the effects of developmental alcohol exposure on circadian rhythms.

## The Structure and Function of the Internal Biological Clock

In mammals, a variety of biological processes, such as the levels of various brain chemicals (i.e., neurotransmitters), blood hormone concentrations, and sleep-wake behavior, undergo rhythmic fluctuations that closely parallel the time course of the daily solar cycle. This temporal organization of mammalian biology to approximate the 24-hour cycle of change in the physical environment occurs even in the absence of external time cues; consequently, these rhythms have been termed circadian. In mammals, including humans, the internal biological clock is responsible for the generation of all circadian rhythms and their synchronization (i.e., entrainment) to light-dark cycles. During entrainment, mammalian circadian rhythms assume not only the periodicity of the light-dark cycle, but also adopt a specific phase relationship to this environmental cycle. Structurally, the internal clock is discretely localized within the brain in paired clusters of nerve cells (i.e., neurons) called the suprachiasmatic nuclei (SCN). Consistent with their circadian timekeeping function, these cells rhythmically release certain brain chemicals (i.e., neuropeptides), such as brain-derived neurotrophic factor (BDNF) and arginine vasopressin (AVP). These neuropeptides as well as other peptides (e.g., vasoactive intestinal polypeptide [VIP]), in turn, may act on other brain regions or peripheral organs to generate a variety of overt circadian rhythms, such as the sleep-wake cycle (see figure).

The normal circadian timekeeping function of the SCN is crucial for maintaining human health and performance by providing for the temporal coordination of internal physiological processes with each other and with the daily light-dark cycle. Damage to the SCN, which would compromise this temporal organization, could thus affect the body’s susceptibility to physiological disorders.

To date, the neurobehavioral consequences of many health problems, including developmental alcohol exposure, on the regulation of circadian rhythmicity are virtually unknown. In animal models, complete destruction of the SCN is necessary to abolish circadian rhythmicity, although experimental or natural injury to subgroups of SCN neurons can produce tangible changes in the SCN-mediated regulation of overt physiological and behavioral rhythms. For example, in rats, partial damage to the SCN alters the animals’ circadian activity rhythm and its pattern of entrainment to light-dark cycles (Pickard and Turek 1985).^4^ Specifically, the destruction of some, but not all, SCN cells results in daily onset of activity at earlier times than normal during exposure to constant environmental conditions and during synchronization to a light-dark cycle.

In a similar fashion, the aging process has been shown to affect discrete components of the human and rodent SCN and the brain chemicals released by the SCN (Roozendaal et al. 1987; Swaab et al. 1988; Krajnak et al. 1998). This impact of aging on SCN cellular organization is associated with functional disturbances in the generation and regulation of circadian behavior, which is often reflected in the sleep-wake cycles of both humans and rodents (Ingram et al. 1982; Moore 1991). For example, aging-related changes can occur in many properties of sleep-wake rhythmicity, including circadian period, entrainment to light-dark cycles, and resetting or phase-shifting to earlier or later times by light (Pittendrigh and Daan 1974; Schwartz 1993), although the reported effects and their relative magnitudes differ considerably between studies. The amplitude of the sleep-wake rhythm also decreases during aging such that day-night differences in activity levels are diminished. Another change in sleep-wake rhythmicity that occurs with advancing age is that sleep becomes more fragmented, resulting in increased arousals and time awake during the night and in a reduction in the total amount of deep sleep. In humans and rodents, these age-related perturbations in the circadian regulation of behavior are accompanied by the partial loss of various neuropeptide-releasing cell types in the SCN and by a damping of the daily peak in SCN levels of these neuropeptides (Roozendaal et al. 1987; Swaab et al. 1988; Krajnak et al. 1998).

## Alcohol’s Potential Influences on the Internal Clock

Based on the well-established evidence that alcohol exposure during rapid brain growth causes cell loss, alters information pathways between brain regions, and decreases the production of brain chemicals responsible for the communication among neurons, it is reasonable to speculate that alcohol may also adversely affect SCN development. Such alcohol-induced insults during the period of rapid brain growth could produce cell loss and/or structural alterations in the SCN that could result in subsequent disturbances of the circadian timekeeping function. Although it is unlikely that the damage to the SCN would be severe enough to cause complete loss of circadian rhythmicity, more subtle disturbances might occur. For example, it is possible that developmental alcohol exposure could lead to permanent reductions in rhythm amplitude, changes in circadian period, and modulation of the SCN clock’s responses to light signals. These potential effects of developmental alcohol exposure on SCN timekeeping function could account for some of the behavioral disturbances observed in FAS patients. For example, depression has been identified as a behavioral manifestation of FAS and circadian rhythm abnormalities have been associated with bipolar affective disorder (i.e., manic-depressive illness) (Moore 1991; Schwartz 1993).

Perturbations in the normal periodicity of circadian rhythms and their synchronization to light-dark signals, whether caused by developmental alcohol exposure or other factors, would be expected to affect chronotherapeutic phenomena in the pharmacologic treatment of diseases, because the efficacy and side effects of many drugs are known to depend on the time of administration in relation to normal body rhythmicity.^5^ Moreover, such perturbations could exacerbate sleep disturbances associated with normal aging and affective disorders, and impair performance in shift workers (Schwartz 1993). Therefore, people with FAS who suffered brain damage within the SCN as a result of developmental alcohol exposure could experience such problems throughout their adult life.

Developmental alcohol exposure could interfere with the circadian clock and its timekeeping function at two levels (see figure). First, it could damage the clock mechanism itself by affecting the structure or function of cells in the SCN. In this case, the changes in circadian function would reflect either a global loss of SCN cells or alterations in the rhythmic activation (i.e., expression) of specific genes that are essential “gears” of the clockworks, such as the *Period* and *Cryptochrome* genes, without which the clock does not work.

Second, developmental alcohol exposure could interfere only with the “hands” of the clock—that is, with the SCN output signals (e.g., the neuropeptides BDNF, AVP, and VIP) and with overt circadian rhythms throughout the body that are regulated by these signals (e.g., the sleep-wake cycle). It will be important to examine the SCN of adults with FAS for evidence of developmental alcohol-induced cell loss, perturbations in the expression of clock genes, and/or alterations in neuropeptide output signals in order to determine the levels at which developmental alcohol could affect circadian rhythms. Once more information becomes available on the severity of the effects of developmental alcohol exposure on circadian clock function and on the mechanism by which alcohol-induced circadian disturbances occur, it may be possible to develop therapies to treat specific rhythm abnormalities.

Studies in rats using alcohol exposure regimens that are equivalent to third-trimester alcohol exposure in humans are beginning to provide specific evidence for the impact of developmental alcohol exposure on SCN rhythmicity and circadian behavior as described in the following paragraph. Furthermore, alcohol exposure has been shown to decrease the levels of a class of growth factors called neurotrophins, which includes BDNF, in other regions of the developing rat brain (Maier et al. 1999). Because heavy maternal alcohol consumption during human pregnancy has been linked to disturbances in the offspring’s sleep-wake patterns (Rosett et al. 1979; Stratton et al. 1996), these observations suggest that developmental alcohol exposure might disrupt the rhythmic activity of the SCN and neuropeptide release and interfere with circadian behavior patterns.

To further explore this hypothesis, Earnest and colleagues (1997) examined the long-term effects of early postnatal alcohol exposure on circadian neurotrophin expression in the SCN and on the rhythm of locomotor activity in rats. Preliminary findings demonstrate that adult rats exposed to alcohol during this critical period of brain development exhibited a shortened circadian sleep-wake cycle, so that their onset of activity occurred much earlier each day. In addition, the activity of animals exposed to developmental alcohol was more fragmented, with frequent alternation between short intervals of sleep and waking. This alcohol-induced change in circadian behavior was associated with marked damping of the BDNF rhythm in the SCN so that peak levels during the night were greatly reduced. Because BDNF has been implicated in the regulation of SCN clock responses to light (Liang et al. 2000), the disruption of BDNF rhythmicity induced by developmental alcohol exposure may also interfere with the light-mediated control of circadian rhythms. Based on these observations, further analysis is warranted to characterize fully the effects of developmental alcohol exposure on circadian timekeeping and their association with damage to the SCN clock or its associated neuropeptides.

## Conclusions

Although the analysis of the relationship between developmental alcohol exposure and circadian rhythmicity is still highly speculative, this research may hold potential in the analysis and treatment of alcohol’s deleterious effect on the developing organism. For example, many behaviors are regulated by numerous brain areas, making it difficult to study alcohol’s effects on those behaviors. In contrast, the discrete localization of circadian clock function in the SCN provides a unique opportunity to identify specific alcohol-related neurobehavioral disturbances and examine their relation to the permanent effects of developmental alcohol exposure on other brain regions. Furthermore, information on the long-term behavioral consequences of FAS is still limited; many characteristics of FAS are not always fully evident in adulthood. Consequently, the identification of permanent alcohol-related alterations in circadian timekeeping may ultimately be useful as a long-term index or behavioral marker for FAS. Finally, research on how the SCN and its circadian function are affected by developmental alcohol exposure could yield important information on the basic mechanisms underlying alcohol-induced injury during rapid brain growth. Such information also could lead to new strategies in the treatment of known behavioral correlates of FAS, such as depression and sleep-wake abnormalities, that result from a disruption of the internal timing of body processes.

## Figures and Tables

**Figure:**
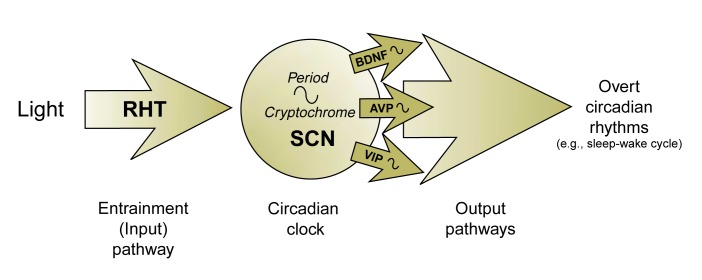
Functional components of the internal timekeeping system responsible for the regulation of circadian, or 24-hour, rhythms in mammals. The central circadian pacemaker, or “clock,” is located in the brain in the suprachiasmatic nuclei (SCN). These cells contain the “gears” that are necessary for accurate timekeeping. These gears consist of certain genes (e.g., the *Period* genes and the *Cryptochrome* genes) that are rhythmically expressed and regulate ciracadian output pathways. These pathways involve various signals that emanate from the SCN, including neuropeptides, such as brain-derived neurotrophic factor (BDNF), arginine vasopressin (AVP), and vasoactive intestinal polypeptide (VIP). These neuropeptides and other output signals act on other brain regions or peripheral organs and result in the generation of overt rhythms in biochemical, physiological, and behavioral processes throughout the body. The retinohypothalamic tract (RHT) is an entrainment, or input, pathway that mediates the synchronization of the circadian clock to the light-dark cycle.

## Footnotes

1 The anterior cerebellum is involved in motor coordination and maintenance of posture.

2 The corpus callosum is a bundle of nerve fibers that connects the two cerebral hemispheres and mediates information transfer between those hemispheres.

3 The hippocampus is involved in learning and memory.

4 In nocturnal rodents, activity levels typically rise near the onset of the dark phase of the daily cycle, whereas rest periods occur primarily during the light phase.

5 For example, chemotherapy agents are usually more effective in treating cancer and are associated with reduced side effects when they are administered in a timed or circadian pattern.
